# Recurrent bacteremia with *Helicobacter cinaedi*: case report and review of the literature

**DOI:** 10.1186/1471-2334-6-86

**Published:** 2006-05-23

**Authors:** Ilker Uçkay, Jorge Garbino, Pierre-Yves Dietrich, Béatrice Ninet, Peter Rohner, Véronique Jacomo

**Affiliations:** 1 Division of Infectious Diseases, Department of Medicine, University Hospital of Geneva, Switzerland; 2 Division of Oncology, Department of Medicine, University Hospital of Geneva, Switzerland; 3 Central Laboratory of Bacteriology, University Hospital of Geneva, Switzerland

## Abstract

**Background:**

*Helicobacter cinaedi *is a rare pathogen in humans, occurring mostly in immuno-compromised patients, with a high potential for recurrence. We describe a case of a patient with lymphoma hospitalized for chemotherapy.

**Case presentation:**

At admission, the patient presented with an indolent and non-prurigenic macular rash around her implantable venous access device. Gram staining of blood cultures revealed the presence of spiral-shaped gram-negative rods that could not be grown upon subculture. *Helicobacter cinaedi *was identified by PCR. No other symptoms or pathology were observed in a whole body CT scan. The implantable venous access device was removed and empiric therapy by ceftriaxone and gentamicin for 2 weeks was initiated, followed by peroral clarithromycin 2 × 500 mg/day and later by levofloxacin 2 × 500 mg/day for 7 weeks. Oncologic remission was achieved 3 months later. However, the patient was re-hospitalized 2 months later for fever, shivering, reappearance of the macular non-prurigenic rash, diarrhea, cough and asthenia. Blood cultures grew *H. cinaedi*. Multiple investigations could not identify the source. Empiric antibiotic therapy of ceftriaxone and doxycycline was started for 2 weeks with resolution of symptoms, followed by an oral combination of amoxicillin, metronidazole and doxycycline for 2 months; doxycycline was continued for another month. Bacteremia has not recurred for a period of 19 months.

**Conclusion:**

Although *H. cinaedi *is considered to be a low virulent bacteria, its potential to cause recurrent bacteremia should not be underestimated. *H. cinaedi *could have an endovascular source of infection and should be treated for an adequate duration with combined antibiotherapy.

## Background

Morphological and genotype studies have shown that different *Helicobacter *species are involved in animal infections. Few cause diseases in humans [1].

*Helicobacter *species have been isolated from the stomach of various mammals, including dogs, cats, ferrets, pigs, monkeys and cheetahs, all of which are associated with various degrees of gastritis in their hosts. *Helicobacter *species have also been isolated from the intestinal tracts of humans, animals, and birds [2,3]. The isolation of *Helicobacter cinaedi *from the colon, liver, and mesenteric lymph nodes of monkeys confirmed its association with hepatitis and colitis in monkeys [4].

Recent studies suggest that *Helicobacter *spp. are involved in cholesterol gallstones in mice and perhaps in humans [5].

*Helicobacter cinaedi*, formerly named *Campylobacter cinaedi*, is an unusual species in some reported series in the literature [6]. This pathogen is found predominantly in severely immuno-compromised patients, such as cancer or AIDS patients. It often causes a non-lethal disease with a high potential for recurrence, requiring long antibiotic therapy for several months. *H. cinaedi *is difficult to grow on traditional culture media. In the majority of cases, diagnosis is possible by PCR. The source of infection is assumed to be intestinal, the hosts are a wide range of animals, especially hamsters. Asymptomatic carriage and infection in immuno-competent patients also occur.

In this report we describe a case of recurrent bacteremia with skin involvement in a patient undergoing chemotherapy; this is a rare clinical presentation. The clinical aspects, therapy pitfalls, a review of the literature and microbiological difficulties are discussed.

## Case presentation

A 53-year-old Caucasian woman without past medical history was hospitalized in August 2003 in our institution because of crippling ischialgic pain in her left leg. Investigations revealed a lesion in the left paravertebral and epidural region of the lumbar spine infiltrating the ipsilateral psoas without metastasis.

Because of exacerbated pain, the appearance of pariesis (difficulty with dorsal flexion of the foot) in her left leg and a large number of differential diagnosis, resection of the paravertebral mass was performed and a portacath was inserted. Histological examination showed a large B-cell lymphoma stage IE. Four cycles of chemotherapy were scheduled with cyclophosphamide, doxorubicin, vincristine and prednisolone in association with methotrexate and monoclonal anti-CD20 antibody. The patient tolerated the first cycle well; agranulocytosis appeared for 4 days (neutrophil count 180 g/l). The leg paresis subsided completely.

At admission for the second cycle of chemotherapy in September 2003, a transient pink macular rash appeared around the skin orifice of the implantable venous access device. No other clinical symptoms were present. Laboratory examinations revealed a C-reactive protein (CRP) of 29 mg/l and a left shift in the leukocyte count of 8%. This slight inflammation could not be explained by the lymphoma alone since previous CRP values were within the normal range. Blood cultures were performed. The rash disappeared spontaneously and the patient became asymptomatic.

Blood cultures were performed using an automated blood culture system (Bactec 9240 Becton Dickinson Diagnostic Instrument Systems, Sparks, MD, USA).

**Figure 1:**
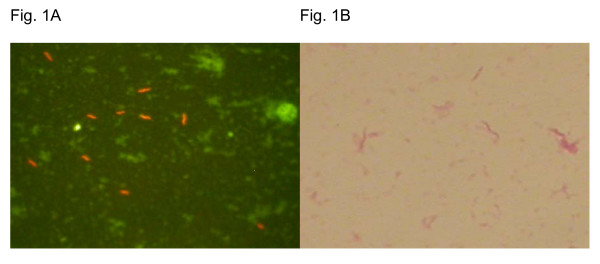
Figure 1A: Acridine Orange staining: spiral bacterium. Figure 1B: Gram-negative rods.

A positive aerobic blood culture vial was indicated after 3 days of incubation. Anaerobic cultures were negative. Gram staining was negative, but Acridine Orange staining revealed a spiral bacterium (figure 1A). Subculture of the positive vials was performed on blood agar plates and chocolate media incubated in a micro-aerobic (5% O_2_, 5 to 10% H_2_) and an anaerobic atmosphere at 35°C, generated with incubation jars in atmosphere generators (GENbox, bioMèrieux SA, Lyon, France). All subcultures remained negative after 5 days, therefore PCR and subsequent sequencing of the 16S rRNA gene was performed on the positive broth. The definitive identification of *H. cinaedi *was achieved by sequencing the product of PCR based on the data of 16S rRNA comparing the nucleotide sequence homology with the GenBank database, where a 99.6% homology was found with *H. cinaedi *(accession number AF 426158). We used the following primer sequences: Primer b 162 5' GAG AGT TTG ATC XTG GCT CAG 3' (Microsynth GmBH) (-20°C). Primer BR-16SR 5' CGC TCG TTG CGG GAC TTA A 3'(Microsynth GmBH) (-20°C).

Additional blood cultures indicated the presence of the same micro-organism and empiric antibiotic therapy with intravenous ceftriaxone 1 × 2 g/day and a catheter lock technique with gentamicin 5 mg/day was administered for 2 weeks.

Despite significant regression of all inflammatory parameters, control blood cultures remained positive for the same organism. The implantable venous access device was removed since an infection of the central vascular access was suspected in absence of another clinical symptoms. Cultures of the implantable venous access device remained negative but bacteremia persisted for another 4 days suggesting another source. However, no other source was found. A whole body computer tomography (CT) scan did not reveal any suspect lesions. Magnetic resonance imaging (MRI), performed due to a headache, revealed an aneurysm, 8 mm in diameter, in the left posterior communicating artery (P2-P3). Coiling was performed with the placement of a detachable platinum coil, believed to be less susceptible to infections than other metals. Discussion arose whether this aneurysm could be of mycotic or congenital origin. No biopsy for histological analysis could be obtained and no previous cerebral images existed. The presence of an aneurysm in a region known for congenital aneurisms led us to diagnose a congenital origin.

After the intravenous treatment, an oral regimen of clarithromycin 2 × 500 mg/day followed by levofloxacin 2 × 500 mg/day was administered for 7 weeks.

The patient underwent three cycles of chemotherapy and 40 Gy of radiotherapy. In January 2004, the patient was considered to be in complete remission with good clinical evolution and she returned to work; the only complaint was symmetric arthralgia in the elbows and knees of unknown origin.

However, in March 2004, the patient was re-hospitalized due to a fever (39°C), shivering, watery diarrhea, vomiting, fatigue, unproductive cough, and a diffuse, red, macular, patchy cutaneous rash all over the body with the exception of mucosal surfaces. The initial suspicion of a viral infection was revised 5 days later when a spiral gram-negative rod was detected in the blood cultures. The CD4 count revealed 290 cells/mm^3 ^attributed to her chemotherapeutic immuno-compromised state. During this second episode of bacteremia, positive blood cultures again revealed a spiral, helical gram-negative rod (figure 1B). This time, subcultures were successful; colonies grew on blood agar incubated in a micro-aerobic (5% O_2_) atmosphere. Again, the identification of *H. cinaedi* was obtained by PCR and sequencing of the 16S rRNA gene.

Minimal inhibitory concentrations (MIC) of potentially active antimicrobials were determined on blood agar incubated micro-aerobically at 35°C with the E-test method for clarithromycin, ciprofloxacin, erythromycin, trimethoprim-sulfamethoxazole, metronidazole and amoxicillin. Interpretation of susceptibility in vitro was based on the NCCLS guidelines for *H. pylori *for clarithromycin, and on published reports for metronidazole and amoxicillin [7,8]. For the other antibiotics, the interpretation was based on the NCCLS guidelines for gram-negative bacilli. The strain was susceptible to amoxicillin (MIC = 0.75 mg/l) and to metronidazole (MIC = 0.25 mg/l), intermediate to clarithromycin (MIC = 3 mg/l) and resistant to the other antibiotics tested (erythromycin, trimethoprim-sulfamethoxazole and ciprofloxacin).

After 2 weeks of successful intravenous ceftriaxone 1 × 2 g/day and peroral doxycycline 2 × 100 mg/day therapy, this regimen was changed to a peroral outpatient treatment. The patient received doxycycline 2 × 100 mg/day, metronidazole 3 × 500 mg/day and amoxicillin 3 × 750 mg/day for 2 months followed by doxycycline alone for a third month, following an empiric eradication therapy for *Helicobacter pylori*. The patient had a good laboratory and clinical response.

Multiple examinations were repeated in order to detect the possible source of the recurrent infections, such as whole body CT scan, transesophageal echocardiography, bacterial stool cultures, colonoscopy with multiple biopsies, bone scintigraphy and cerebral angiographic MRI. No pathology, no recurrent neoplastic disease or cerebral aneurysms were found. An unspecific inflammation was seen in the colonoscopy without histological evidence of bacterial infection. This observation was attributed to the patient's post-irradiation status. Further investigations were abandoned due to the favorable progress under antibiotic therapy. The source of the recurrent infection could not be identified but was presumed to be either of endovascular or intestinal origin.

In November 2005, 19 months after the recurrence, the patient is in complete remission and in good clinical condition.

## Discussion

Like other non-pylori *Helicobacters*, *H. cinaedi *is an unusual pathogen in humans and difficult to culture with the normal routine procedures. Since the first cases of proctocolitis in male AIDS patients were described in 1985 [9], several other cases have been reported in the literature. Asymptomatic carriage certainly occurs. The micro-organism, considered to be enterohepatic, seems to have a low degree of virulence. It is regarded as an opportunist. Recently, Taylor et al. [10] reported for the first time an important putative virulence factor in *H. cinaedi *confirmed by the production of cytolethal distending toxin, which causes distention in cells and arrest in the G2/M phase of cell division. Also motility by means of flagella is generally regarded as a virulence determinant in *Helicobacter *and *Campylobacter *species. *H. cinaedi *shares the production of cytolethal distending toxin with other enteric pathogenic species. *H. cinaedi *may cause symptomatic diseases in immuno-compromised patients, such as those with HIV, cancer or after transplantation [9,11-18].

Rare cases of disease in immuno-competent patients have been reported [19,20]: a patient with bacteremia and arthritis [19] or erysipelas [20], several patients with recurrent fever and rash [11], and in neonates [21]. Interestingly, all immuno-competent patients and neonates with *H. cinaedi *disease had been in contact with animals [19]. Other cases of bacteremia in immuno-compromised patients after renal failure [22] and X-linked agammaglobulinemia [23] have also been published in the literature.

The route of infection is rarely known, and presumed to be oral. As a reservoir, *H. cinaedi *has been identified in many animals, for example, rats, hamsters, dogs, cats, foxes, poultry, wild birds, and monkeys. Hamsters, in particular, are known to be a common natural reservoir [24]. The role of the micro-organism in these animal hosts is unknown. Interestingly, our patient's daughter, living in the same household, fed her pet snake with rats. The snake died several months before the mother's illness. Perhaps both mother and daughter have been asymptomatically infected for a long time by the rats the snake was fed with. Rats are known to be carriers of *H. cinaedi *[25]. Symptoms could have emerged in the mother after immuno-suppressive chemotherapy. We assume that the patient would not have experienced the *H. cinaedi *infection without the associated lymphoma and the chemotherapy. Cultures from the daughter were not possible to perform.

*H. cinaedi *infections may present various clinical manifestations, ranging from proctocolitis [9], gastroenteritis [26], meningitis in neonates [21], localized pain [27], rash, or bacteremia [13]. The more immuno-compromised the patients, the more severe are the symptoms. Nevertheless, Kiehlbauch et al. [11] reported that two symptoms are predominant, namely fever and rash [11]. Our case presented both symptoms, whereas the additional symptoms of diarrhea and arthralgia are less frequently reported [11]. In our case, *H. cinaedi *could not be isolated from the colonoscopic biopsies and stool cultures. Other infectious agents could not be identified in the stool cultures.

The origin of the first bacteremia in our case remains unknown but we have assumed it to be the gastrointestinal tract since the mucosal cells have been damaged by combined chemo- and radiotherapy.

Our investigations revealed a cerebral aneurysm of the posterior communicating artery. Its localization and its single status indicate a possible congenital origin. No new aneurysms or change in size were observed during the second episode of bacteremia. A whole body angiography of the large arteries did not reveal an endovascular origin.

*H. cinaedi *is a fastidious organism, rendering microbiological diagnosis difficult. It rarely grows on traditional culture media [25]. At best, growth may be obtained on rich, non-selective media (blood or chocolate agar) incubated in a micro-aerobic (5% O_2_) atmosphere at 35°C [11]. The diagnosis is mainly established by gene amplification techniques such as PCR and subsequent sequencing [28]. With these techniques, however, antimicrobial susceptibility in vitro cannot be determined.

No clear guidelines are available in the literature concerning the choice or duration of antibiotic therapy. Many antibiotic agents, alone or in combination, have been successfully used, such as penicillin, ampicillin, cefazolin, erythromycin, ciprofloxacin, aminoglycosides, tetracyclines and rifampicin [11,19,21,29]. A large review of 23 cases of bacteremia reported that penicillins, tetracycline, and aminogylcosides are more effective than cephalosporins, erythromycin, or ciprofloxacin. Erythromycin resistant *H. cinaedi *has also been identified [30]. Quinolones alone may not completely eradicate *H. cinaedi*, which explains the frequent reports of recurrent disease after quinolone monotherapy [11].

The reported duration of antibiotherapy for *H. cinaedi *bacteremia ranges from 10 days to 12 weeks [19,29]. Due to the frequent recurrences, prolonged antibiotic treatment is necessary [11-13]. In our case, a 2-week course of combined antibiotherapy with ceftriaxone and gentamicin, followed by 5 weeks of peroral clarithromycin and levofloxacin was not able to eradicate the micro-organism despite its susceptibility in vitro. Other possible reasons that could explain the recurrent illness were excluded, such as malabsorption, suspected malcompliance or macroscopic intestinal lesions. We therefore chose to treat the second recurrent episode for a sufficiently long 3-month period; so far it has been successful.

## Conclusion

In conclusion, we report that recurrent bacteremia due to *H. cinaedi *without any anatomic lesion may occur; the micro-organism may re-emerge and prolonged antibiotic treatment is necessary. Optimal antimicrobial treatment and its duration remain to be established.

## Competing interests

The author(s) declare that they have no competing interests.

## Authors' contributions

IU and JG drafted and wrote the final manuscript, interpreted the data and made the review of the literature. IU and JG contributed equally to this manuscript.

PYD contributed to the oncological part and followed the clinical evolution of the patient.

BN, PR and VJ performed the laboratory analysis and contributed to the microbiological part.

*All authors read and approved the final manuscript*.

*There was no funding for this manuscript*.

## Pre-publication history

The pre-publication history for this paper can be accessed here:


